# Antenatal Exposure to UV‐B Radiation and Preeclampsia: A Retrospective Cohort Study

**DOI:** 10.1161/JAHA.120.020246

**Published:** 2021-06-22

**Authors:** Claire E. Hastie, Daniel F. Mackay, Tom L. Clemens, Mark P. C. Cherrie, Lauren J. Megaw, Gordon C. S. Smith, Sarah J. Stock, Chris Dibben, Jill P. Pell

**Affiliations:** ^1^ Institute of Health and Wellbeing University of Glasgow United Kingdom; ^2^ Centre for Research on Environment, Society and Health School of Geosciences University of Edinburgh United Kingdom; ^3^ King Edward Memorial Hospital for Women Subiaco Australia; ^4^ Department of Obstetrics and Gynaecology University of Cambridge United Kingdom; ^5^ Usher Institute University of Edinburgh United Kingdom; ^6^ Institute of Geography University of Edinburgh United Kingdom

**Keywords:** environmental exposures, preeclampsia, seasonal variations, UV light, Risk Factors, Preeclampsia

## Abstract

**Background:**

Risk of preeclampsia varies by month of delivery. We tested whether this seasonal patterning may be mediated through maternal vitamin D concentration using antenatal exposure to UV‐B radiation as an instrumental variable.

**Methods and Results:**

Scottish maternity records were linked to antenatal UV‐B exposure derived from satellites between 2000 and 2010. Logistic regression analyses were used to explore the association between UV‐B and preeclampsia, adjusting for the potential confounding effects of month of conception, child's sex, gestation, parity, and mean monthly temperature. Of the 522 896 eligible singleton deliveries, 8689 (1.66%) mothers developed preeclampsia. Total antenatal UV‐B exposure ranged from 43.18 to 101.11 kJ/m^2^ and was associated with reduced risk of preeclampsia with evidence of a dose‐response relationship (highest quintile of exposure: adjusted odds ratio, 0.57; 95% CI, 0.44–0.72; *P*<0.001). Associations were demonstrated for UV‐B exposure in all 3 trimesters.

**Conclusions:**

The seasonal patterning of preeclampsia may be mediated through low maternal vitamin D concentration in winter resulting from low UV‐B radiation. Interventional studies are required to determine whether vitamin D supplements or UV‐B–emitting light boxes can reduce the seasonal patterning of preeclampsia.

**Nonstandard Abbreviations and Acronyms**
25(OH)D: 25‐hydroxy vitamin D

**Clinical Perspective**

**What Is New?**

- We linked an environmental exposure, UV‐B radiation, during pregnancy with risk of preeclampsia.
- Our hypothesis was that this exposure may explain the seasonal patterning of preeclampsia.
- We observed an inverse relationship between antenatal UV‐B exposure and risk of preeclampsia.

**What Are the Clinical Implications?**

- The use of vitamin D supplements, or UV‐B–emitting light boxes, in high latitude countries could eliminate seasonality.
- Intervention studies can assess this.

There is growing evidence of seasonal patterning in the risk of preeclampsia. A population‐wide study of deliveries in Norway^1^ and subsequent systematic review^2^ observed that the risk of preeclampsia was highest in pregnancies resulting in winter deliveries. Rylander and Lindqvist^3^ reported that, in Sweden, the incidence of eclampsia nearly doubled for births in winter compared with other seasons. In Texas, half the distance from the equator, the incidence is still highest among winter deliveries but the magnitude of seasonal variation in preeclampsia and eclampsia is less.^4^ Since gestational age at delivery can vary widely, month of delivery is a poor measure of the season within which each trimester occurs. Therefore, month of conception is a better measure of exposure to environmental factors at critical periods of pregnancy, and there is some evidence that season of conception is more strongly associated with preeclampsia than season of delivery, with 70% higher odds of preeclampsia following conceptions in summer.^2^, ^5^, ^6^, ^7^

It is plausible that vitamin D may play a role in this seasonal patterning. Case‐control studies have found significantly lower circulating 25‐hydroxy vitamin D (25(OH)D) concentrations in women with preeclampsia and eclampsia, compared with healthy, normotensive pregnant women.^8^, ^9^, ^10^, ^11^ Evidence from cohort studies suggests that the third trimester may be a critical phase for vitamin D production. It has been estimated that UV exposure in the third trimester explains 40% of maternal 25(OH)D.^12^ Two cohort studies that measured maternal 25(OH)D before 20 weeks' gestation^13^, ^14^ did not find associations with preeclampsia. Wei et al^15^ found an association with 25(OH)D concentration at 24 to 26 weeks' gestation but not at 12 to 19 weeks' gestation. Similarly, Bärebring et al^16^ found that 25(OH)D concentrations in trimester 3, but not trimester 1, were inversely associated with preeclampsia. An association between vitamin D concentrations late in pregnancy and preeclampsia has been corroborated by systematic reviews of observational studies,^17^, ^18^ and the most recent meta‐analysis of 27 trials of vitamin D supplementation, comprising 4777 participants, reported a reduced risk of preeclampsia (odds ratio, 0.35; 95% CI, 0.26–0.52) with evidence of a dose relationship.^19^ A recent Cochrane review concluded that there is moderate certainty that supplementation with vitamin D and calcium during pregnancy probably reduces the overall risk of preeclampsia by around a half, based on 4 trials comprising 11 174 women^20^ but did not explore the issue of seasonal variations.

Vitamin D is produced in response to UV‐B exposure. The amount of UV‐B reaching the earth's surface is highest in summer months and, in winter, is usually too low to stimulate vitamin D production in high‐latitude countries. UV levels are strongly correlated with season, but superimposed on this annual cycle there is an 11‐year solar cycle over which changes in the sun's oscillatory magnetic field result in variations in the number of sunspots and, therefore, solar radiation. As a result, UV levels in a given season vary between years; therefore, adjusting for month of conception can disentangle associations with UV from associations with other seasonally patterned environmental and behavioral phenomena.

Our study aim was to explore whether antenatal UV‐B exposure was associated with preeclampsia incidence, independent of month of conception, among all singleton children born in Scotland over an 11‐year period.

## Methods

The data sets analyzed during the current study are available in the National Services Scotland National Safe Haven, https://www.isdscotland.org/Products‐and‐Services/eDRIS/Use‐of‐the‐National‐Safe‐Haven/.

All methods were carried out in accordance with the EU General Data Protection Regulation. All experimental protocols were approved by National Health Service Scotland's Public Benefit and Privacy Panel for Health and Social Care on December 5, 2019 (Reference 1617‐0001). Informed consent waived by National Health Service Scotland's Public Benefit and Privacy Panel for Health and Social Care.

We linked routine maternity records on singleton infants delivered in Scotland between 2000 and 2010 inclusive to environmental databases on UV solar irradiance and temperature. The Scottish Morbidity Record 02 records the child's sex and date of birth, gestation at delivery, whether it was a multiple delivery, and any maternal diseases. Preeclampsia was defined as *International Classification of Diseases, Ninth Revision* (*ICD‐9*) codes 642.4 or 642.5, or *International Classification of Diseases, Tenth Revision* (*ICD‐10*) codes O14.0, O14.1, or O14.9. Since the early 1990s, gestational age at delivery has been confirmed by ultrasound conducted in the first half of pregnancy in >95% of pregnant women in the United Kingdom. If there is more than 7 days' difference between gestational age calculated from ultrasound and from last menstrual period, the former is used.^21^ Date of conception was derived from date of delivery minus gestational age at delivery plus 2 weeks. Trimester 1 comprised the first 3 months of pregnancy from month of conception; trimester 2 comprised months 4, 5, and 6; and trimester 3 comprised all subsequent months until delivery.

Global 5‐km UV radiation irradiance data were produced by the Japan Aerospace Exploration Agency using measurements from the moderate resolution imaging spectroradiometer instrument onboard the National Aeronautics and Space Administration's aqua and terra satellites. Downward irradiance values (ie, combined direct and diffuse radiation on a horizontal plane) for UV‐B (280–315 nm) was available at daily resolution from 2000 to 2010 inclusive. We downloaded UV‐B values, between January 1, 2000, and December 31, 2010, for each latitude and longitude within the Great Britain bounding box (48°N to 63°N; −11°W to 4°E) from the publicly accessible Japan Aerospace Exploration Agency FTP site (ftp://apollo.eorc.jaxa.jp/pub/JASMES/Global_05km/). The Great Britain points were then interpolated, via inverse weighted distance, to raster images. The rasters were projected to Ordinance Survey Great Britain 1936/British National Grid. Finally, we took the Northing and Easting centroids of each postcode unit in Scotland (Ordnance Survey codepoint ver 2017.4.0) and used these to extract the UV‐B values for only those grid squares covering residential areas in Scotland. Scotland‐wide mean daily values were then calculated for each month resulting in a mean monthly time series of UV‐B irradiance values measured in W/m^2^. These were converted to kJ/m^2^ by multiplying by 86 400 (seconds in the day) and dividing by 1000 to be consistent with other epidemiologic studies. We linked these data to pregnancy records by month and year of conception. UV‐B exposure was calculated over each trimester of pregnancy and total exposure over the whole of pregnancy. These were then categorized into overall and trimester‐specific quintiles (UV‐B method previously described^22^).

Mean monthly air temperatures (°C) for 2000 to 2010 were downloaded from the Centre for Environmental Data Analysis website. These data are derived from the Met Office historical weather observations, which are then spatially interpolated to produce a regular 5‐km grid. The interpolation process takes account of latitude and longitude, altitude and terrain shape, coastal influence, and urban land use.^23^

Since UV data were available for only 2000 to 2010 inclusive, children conceived before 2000 or born after 2010 were excluded as they did not have UV exposure data for the whole of their intrauterine period. We excluded from the study children who were born <24 or >44 weeks' gestation, or had birthweight <400 or >6500 g. These exclusions were made because values outside these ranges are indicative of pregnancy complications that may introduce confounding. We tested whether there was evidence of any association between UV‐B exposure and preeclampsia. A series of multivariate logistic regression analyses were performed of the associations between trimester‐specific and overall UV‐B and preeclampsia. The models were adjusted sequentially for month of conception, then child's sex, estimated gestation and parity, then mean monthly temperature. Month of conception was modeled as a dummy variable. All analyses were undertaken using Stata version 14.

## Results

Overall, 528 175 singleton children were born in Scotland between 2000 and 2010. Of these, 657 were ineligible for inclusion: 35 were born at <24 weeks' gestation; 21 were born at >44 weeks' gestation, 252 had missing data on gestation; 74 had birthweight <400 g; and 275 had birthweight >6500 g. The remaining 527 518 were eligible for inclusion. A further 2 had to be excluded because the mother's age was missing, 25 because the child's sex was missing, and 4595 because parity was missing. Therefore, the final study population comprised 522 896 deliveries. Over the 11‐year study period (2000–2010), mean monthly exposure to UV‐B ranged from 0.51 kJ/m^2^ (range, 0.37–0.69 kJ/m^2^) in December to 19.23 kJ/m^2^ (range, 16.87–21.59 kJ/m^2^) in June. Distribution of conceptions within a month did not vary across months. Therefore, use of a chronologically centered midmonth mean did not introduce bias, although it may have reduced statistical power.

The mother developed preeclampsia in 8689 (1.66%) pregnancies, ranging from 1.55% for October conceptions to 1.75% for February conceptions (Figure). The incidence of preeclampsia was 1.68% for male offspring and 1.64% for female offspring. Preeclampsia occurred in 7.43% of pregnancies resulting in preterm delivery, 1.33% of term and 0.37% of postterm. After adjusting for month of conception, there was a dose‐dependent, inverse relationship between UV‐B exposure over the whole of pregnancy and preeclampsia (Table). This reached statistical significance for UV‐B quintiles 4 and 5, compared with the lowest exposure quintile, and persisted after adjustment for the child's sex, estimated gestation at delivery, parity, and temperature. Preeclampsia was associated with UV‐B exposure in all trimesters.

**Figure 1:**
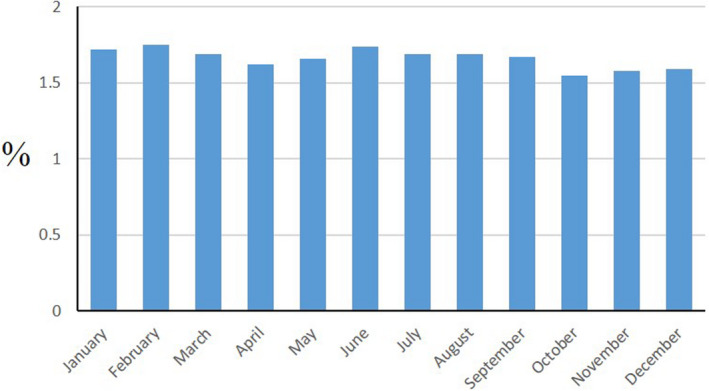
Crude prevalence of preeclampsia by month of conception.

**Table 1:** Association Between Quintile of UV‐B Exposure and Record of Preeclampsia by Trimester and Whole Pregnancy

	Trimester 1		Trimester 2		Trimester 3		Whole Pregnancy	
	OR (95% CI)	*P* Value	OR (95% CI)	*P* Value	OR (95% CI)	*P* Value	OR (95% CI)	*P* Value
Model 1*								
1 (lowest)	Referent		Referent		Referent		Referent	
2	0.940 (0.806–1.097)	0.432	1.027 (0.886–1.191)	0.721	0.817 (0.704–0.949)	0.008	1.119 (0.979–1.278)	0.098
3	0.915 (0.720–1.163)	0.466	0.947 (0.751–1.194)	0.646	0.694 (0.511–0.943)	0.019	0.916 (0.767–1.094)	0.334
4	0.893 (0.659–1.209)	0.463	0.675 (0.513–0.888)	0.005	0.705 (0.498–0.999)	0.049	0.722 (0.583–0.896)	0.003
5 (highest)	0.586 (0.417–0.823)	0.002	0.357 (0.257–0.495)	<0.001	0.547 (0.371–0.805)	0.002	0.526 (0.418–0.661)	<0.001
Model 2^†^								
1 (lowest)	Referent		Referent		Referent		Referent	
2	0.912 (0.780–1.066)	0.248	1.010 (0.869–1.174)	0.895	0.810 (0.696–0.943)	0.007	1.090 (0.952–1.248)	0.214
3	0.898 (0.704–1.146)	0.386	0.888 (0.702–1.123)	0.323	0.688 (0.505–0.938)	0.018	0.867 (0.723–1.038)	0.120
4	0.889 (0.653–1.210)	0.455	0.629 (0.476–0.831)	0.001	0.700 (0.492–0.995)	0.047	0.671 (0.539–0.835)	<0.001
5 (highest)	0.559 (0.396–0.790)	0.001	0.320 (0.229–0.447)	<0.001	0.536 (0.363–0.794)	0.002	0.475 (0.376–0.599)	<0.001
Model 3^‡^								
1 (lowest)	Referent		Referent		Referent		Referent	
2	0.959 (0.819–1.123)	0.603	1.031 (0.887–1.198)	0.695	0.822 (0.706–0.957)	0.011	1.159 (1.009–1.331)	0.037
3	1.033 (0.805–1.325)	0.801	0.916 (0.723–1.159)	0.465	0.705 (0.517–0.962)	0.028	0.961 (0.798–1.158)	0.677
4	1.013 (0.742–1.384)	0.934	0.664 (0.502–0.878)	0.004	0.717 (0.504–1.021)	0.065	0.771 (0.614–0.967)	0.025
5 (highest)	0.662 (0.466–0.940)	0.021	0.347 (0.248–0.486)	<0.001	0.559 (0.377–0.828)	0.004	0.566 (0.442–0.723)	<0.001

OR indicates odds ratio.

* Adjusted for month of conception.

^†^ Also adjusted for child's sex, estimated gestation, and parity.

^‡^ Also adjusted for mean monthly temperature over the trimester/whole pregnancy.

## Discussion

Among 522 896 pregnancies in Scotland over an 11‐year period, exposure to UV‐B was inversely associated with the risk of preeclampsia, with some evidence of a dose relationship. This was independent of month of conception and, therefore, other seasonally patterned phenomena such as diet, physical activity and smoking, as well as the child's sex, gestation, parity, and temperature.

Most vitamin D is produced in the skin through UV‐B exposure. Both season and weather affect the amount of UV‐B reaching the earth's surface. Therefore, in high‐latitude countries, such as Scotland, the amount of UV‐B in winter months is generally insufficient for vitamin D production because of a lower zenith angle and higher cloud cover resulting in a greater amount of atmosphere to penetrate. Vitamin D insufficiency, defined as circulating 25(OH)D concentrations <40 nmol/L, is twice as likely in Scottish residents than people resident in other parts of Britain.^24^

Our findings support our hypothesis that the higher risk of preeclampsia during pregnancies resulting from summer conceptions may be attributable to lower levels of exposure to UV‐B, in the second half of pregnancy, likely mediated via lower levels of maternal vitamin D. Our findings are consistent with a study of solar radiation in an Australian cohort, which found that pregnancy hypertension was positively correlated with solar radiation 1 month after conception but negatively correlated with solar radiation 7 months after conception.^25^ The study measured total solar radiation only, not UV‐B. Furthermore, they did not adjust for any potential confounders. Therefore, the apparent association with solar radiation may instead have simply reflected an association with other seasonally patterned environmental (eg, temperature) and lifestyle (eg, physical activity) phenomena.

This study covered the entire Scottish population nonselectively. We used an existing maternity database that is not collected primarily for research use. However, it is subjected to regular quality‐assurance checks in which a sample of digital records are validated against clinical records, and completeness and accuracy have been consistently high for the variables used in this study.

As well as UV, other environmental exposures such as temperature exhibit seasonal patterning. Furthermore, lifestyle behaviors such as diet and physical activity vary by season. Therefore, we adjusted for outdoor temperature and month of conception to control for other seasonally patterned exposures for which we did not have data. Adjustment for child's sex was a strength because boys have a different seasonal patterning of conception from girls, with male conceptions peaking in autumn and female conceptions in spring.^26^

As with all observational studies, association does not necessarily infer causation since residual confounding is possible. Mendelian randomization provides evidence that low vitamin D concentrations are causally linked to higher blood pressure and hypertension.^27^ Thus far, one small Mendelian randomization study has failed to demonstrate a similar causal link between low vitamin D and preeclampsia.^28^ Larger studies that test more genetic instruments are required. In this study, ambient UV‐B was similarly used an instrumental variable for vitamin D.^12^

We calculated mean daily levels of UV‐B for each month over Scotland as a whole. Greater granularity would have been preferable; however, Scotland measures 729 km (453 miles) from the northernmost to the southernmost settlement, which equates to a relatively small range of latitudes (from 54.4° North to 60.5° North). Our calculation of UV exposure assumed that women resident in Scotland remained in Scotland during their pregnancy. No data were available on changes in UV exposure attributable to holidays and trips abroad. A limitation of Scottish Morbidity Record 02 is that it does not record maternal lifestyle data such as physical activity or weight gain during pregnancy. Similarly, we had no data on individual differences in exposure to UV attributable to the proportion of time spent outdoors, choice of clothing, or use of sunblock.

## Perspectives

We observed an inverse relationship between antenatal UV‐B exposure and risk of preeclampsia. This lends support to the National Institute for Health and Care Excellence guideline that all pregnant women should take 10 µg of vitamin D supplements daily (400 IU/day), and National Health Service guidance on safe sun exposure to produce vitamin D.^29^ However, further corroborative evidence is required that UV‐B is causally linked to the seasonal patterning of preeclampsia, and intervention studies are needed to test whether use of vitamin D supplements or UV‐B emitting light boxes in high‐latitude countries could eliminate seasonality.

## Author contributions

Dr Pell had the original concept. Drs Pell and Mackay obtained maternity data; and Drs Clemens, Cherrie, and Dibben obtained environmental data. Dr Hastie undertook the statistical analyses. Drs Hastie, Pell, Mackay, Clemens, Cherrie, Dibben, Smith, Stock, and Megaw interpreted the results. Drs Hastie and Pell drafted the manuscript. Drs Hastie, Pell, Mackay, Clemens, Cherrie, Dibben, Smith, Stock, and Megaw revised the manuscript and approved the final version.

## Sources of Funding

This work was supported by Health Data Research UK (Edin‐1 to Hastie). Provision of the UV data was funded by a joint Natural Environment Research Council, Medical Research Council, and Chief Scientist Office project grant (NE/P010911/1); Tommy's Charity; and Health Data Research UK funding (Edin‐1). Dr Stock is funded by Wellcome Trust Clinical Career Development Fellowship (209560/Z/17/Z). Funders played no role in conducting the research or writing the paper.

## Disclosures

None.
